# Luminal lncRNAs Regulation by ERα-Controlled Enhancers in a Ligand-Independent Manner in Breast Cancer Cells

**DOI:** 10.3390/ijms19020593

**Published:** 2018-02-16

**Authors:** Valentina Miano, Giulio Ferrero, Valentina Rosti, Eleonora Manitta, Jamal Elhasnaoui, Giulia Basile, Michele De Bortoli

**Affiliations:** 1 Center for Molecular Systems Biology, University of Turin, Orbassano, 10043 Turin, Italy; valentina.miano@unito.it (V.M.); giulio.ferrero@unito.it (G.F.); elhasnaouij@gmail.com (J.E.); 2 Department of Clinical and Biological Sciences, University of Turin, Orbassano, 10043 Turin, Italy; rosti@ingm.org (V.R.); eleonora.manitta@gmail.com (E.M.); 3 Department of Computer Science, University of Turin, 10149 Turin, Italy; 4 Italian Institute for Genomic Medicine (IIGM), 10126 Turin, Italy; giulia.basile@iigm.it

**Keywords:** lncRNA, super enhancer, estrogen receptor, breast cancer

## Abstract

Estrogen receptor-α (ERα) is a ligand-inducible protein which mediates estrogenic hormones signaling and defines the luminal BC phenotype. Recently, we demonstrated that even in absence of ligands ERα (apoERα) binds chromatin sites where it regulates transcription of several protein-coding and lncRNA genes. Noteworthy, apoERα-regulated lncRNAs marginally overlap estrogen-induced transcripts, thus representing a new signature of luminal BC genes. By the analysis of H3K27ac enrichment in hormone-deprived MCF-7 cells, we defined a set of Super Enhancers (SEs) occupied by apoERα, including one mapped in proximity of the *DSCAM-AS1* lncRNA gene. This represents a paradigm of apoERα activity since its expression is largely unaffected by estrogenic treatment, despite the fact that E2 increases ERα binding on DSCAM-AS1 promoter. We validated the enrichment of apoERα, p300, GATA3, FoxM1 and CTCF at both *DSCAM-AS1* TSS and at its associated SE by ChIP-qPCR. Furthermore, by analyzing MCF-7 ChIA-PET data and by 3C assays, we confirmed long range chromatin interaction between the SE and the *DSCAM-AS1* TSS. Interestingly, CTCF and p300 binding showed an enrichment in hormone-depleted medium and in the presence of ERα, elucidating the dynamics of the estrogen-independent regulation of *DSCAM-AS1* expression. The analysis of this lncRNA provides a paradigm of transcriptional regulation of a luminal specific apoERα regulated lncRNA.

## 1. Introduction

Estrogen Receptor α (ERα) is the key transcriptional factor of the luminal breast cancer subtype, which comprises three quarter of breast cancer cases [1], by mediating estrogen signaling in breast cancer cells. Nowadays, current endocrine treatments that block estrogen action include Selective Estrogen-Receptor Modulators (SERMs), Fulvestrant (pure anti-estrogen that degrades ERα protein) and Aromatase Inhibitors (AI) [2]. SERMs are synthetic compounds that function as agonists or antagonists for estrogen receptors in a tissue-specific manner. The first SERM that has been used successfully in the clinic is tamoxifen (TAM) which functions as cell type-specific anti-estrogens [3]. The most common endocrine treatments today are AIs, which block the biosynthesis of estrogens [4]. However, this therapy fails in one-fourth of ERα+ breast cancer cases because of de-novo or acquired endocrine resistance, which is generally characterized by accelerated tumor growth and increased aggressive behavior [5].

ERα regulates biological processes in breast cancer cells by ligand-dependent (estrogen-ERα complex) or ligand-independent action (apoERα) [6]. Whereas estrogen dependent signaling was deeply studied in the last decades [7], few works were based on understanding apoERα mechanism in BC. It was reported that apoERα acts in cooperation with FOXA1 and AP2γ to maintain the luminal epithelial enhancer landscape and when one of these factors are suppressed, enhancers progressively collapse [6]. As previously demonstrated for E2-bound ERα genomic action [8], gene expression regulation by apoERα seems dependent on long range interaction between apoERα-bound enhancers and promoters.

Recently, Super-Enhancers (SEs) emerged as a novel class of distal *cis* regulatory elements, defined as large clusters of putative enhancers in close genomic proximity which drive gene expression to define cell lineage identity [9]. SEs tend to span large genomic regions with unusually strong enrichment of lineage-specific TFs and co-activators binding. These stitched enhancers have additive and synergistic functions allowing them to drive high levels of tissue-specific gene expression. Commonly, SEs were predicted by identification of genomic regions enriched in H3K27ac ChIP-Seq signal compared to input or control experiment [10]. SEs represent the core of the transcription regulation for cell-type-specific gene expression and play a key role in cellular development and dynamic response to environmental stimuli [11,12]. Deciphering the active SEs in a specific context is helpful for the identification of key *cis*-regulation of coding and non-coding genes expression [13]. In MCF-7 cells, it was recently demonstrated that ERα is required for SEs formation upon binding to canonical response elements and in presence of FoxA1 pioneering factor [14]. This study elucidated SEs assembly in an estrogen-dependent manner, but the mechanism of SEs modulation by ligand-independent ERα remains unclear.

A full collection of lncRNAs has emerged during the last decade, stressing on their importance as regulators of cellular development and differentiation. They are expressed at lower levels than protein-coding transcripts, but compared to the latter, their pattern of expression is remarkably cell type-specific [15,16]. Indeed, to achieve a comprehensive annotation, lncRNAs expression has been quantitatively analyzed in several tissues and cell types by high-throughput RNA sequencing (RNA-seq) [17]. In BC research, lncRNAs have been involved in regulation of mammary epithelial cells homeostasis [18] and several studies focused on lncRNAs regulated by estrogen [8,19,20]. Finally, we recently identified for the first time a set of apoERα-regulated lncRNAs [21]. Comparison with publicly available data showed that these lncRNAs are highly specific for the luminal BC subtype and therefore very promising in defining peculiar BC features. Among this set, *DSCAM-AS1* is the most abundantly expressed lncRNA in BC cell lines and tissue samples. Expression of *DSCAM-AS1* has a very peculiar dependence over ERα: the increased ERα binding at *DSCAM-AS1* promoter upon estradiol stimulus does not lead to a concordant up-regulation of gene transcription over at least 24 h indicating a necessary and sufficient apoERα action for the regulation of *DSCAM-AS1* transcription [21]. It has been reported that ERα binding to *DSCAM-AS1* promoter is high in tumor tissues from patients unresponsive to Tamoxifen treatment and that *DSCAM-AS1* expression is upregulated in Tamoxifen-resistant cellular models [22], contrary to typical estrogen-responsive genes that are strongly downregulated. These results suggest that actually distal putative enhancer or SE regions might be responsible for this peculiar transcriptional regulation and might be more generally involved in transcriptional regulation of cell-specific lncRNAs.

In the present study, we extended the analysis of apoERα-regulated transcriptome in MCF-7, characterized apoERα binding at SEs (SE-aERBSs) and defined apoERα-regulated lncRNAs that are associated and regulated by SE activation. In particular, we focused our attention on *DSCAM-AS1* associated SE which is stabilized in a hormone-independent manner and whose regulation depends on the presence of apoERα. It represents a paradigmatic example of how luminal lncRNAs are regulated by specific SEs activated through unliganded ERα, which is extremely important to understand luminal breast tumor growth and progression, in view of the fact that common endocrine treatments today (AIs) deplete the organism of estrogenic hormones.

## 2. Results

### 2.1. A Comprehensive List of apoERα Regulated Genes through SE Binding

**Figure 1:**
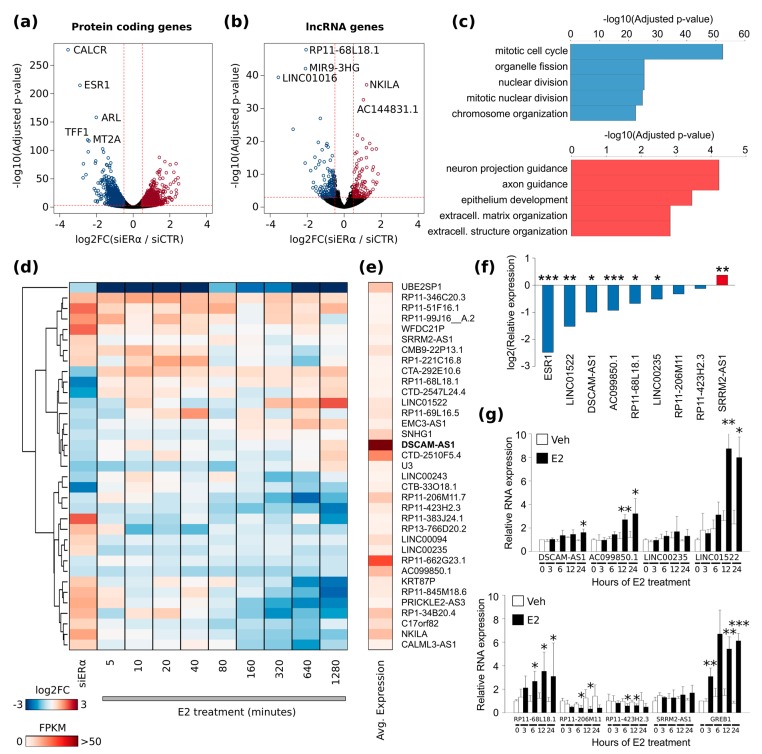
ApoERα regulated genes through SE binding. (**a**,**b**) Volcano plots reporting the adjusted *p*-value and the log2 Fold Change (FC) of (**a**) the protein-coding genes and (**b**) lncRNAs computed from RNA-seq data comparing MCF-7 cells transfected with siERα and siCTR in hormone-deprived (HD) medium; (**c**) Bar plot reporting the -log10 adjusted *p*-value of the most significant Gene Ontology Biological Processes enriched for the genes down-regulated (**top**) or up-regulated (**down**) upon ERα silencing in HD; (**d**) Heat map illustrating differential expression of Super-Enhancer (SE)-associated lncRNAs upon siERα transfection and upon nine-hours E2 treatment; (**e**) Heat map illustrating expression values of SE-associated lncRNAs as FPKM; (**f**) Bar plot reporting the log2 relative expression of ERα mRNA and eight candidate SE-associated lncRNAs upon ERα silencing in HD; *p*-value of three biological replicates by unpaired t-test: * *p* < 0.05; ** *p* < 0.01; *** *p* < 0.001; (**g**) Bar plot reporting the relative expression of eight candidate SE-associated lncRNAs and GREB1 (positive control) in a time-course experiment of 17β-estradiol (E2) or vehicle (Veh) treatment; SD of three biological replicates; *p*-value by unpaired t-test: * *p* < 0.05; ** *p* < 0.01; *** *p* < 0.001.

To define a comprehensive set of apoERα gene targets, we performed a long RNA-Seq analysis in triplicate of MCF-7 cultured in Hormone Deprived medium (HD) for 96 h and transfected with control siRNA (siCTR) or against ERα (siERα). Differential expression analysis revealed a set of 2487 differentially expressed (DE) coding and non-coding genes of which 1376 were down-regulated and 1110 up-regulated upon ERα silencing (Supplementary Table S1). Excluding *ESR1*, the most significant down-regulated protein coding genes were *CALCR*, *ARL1*, and *TFF1* while *KLF6*, *ZNF469*, and *TP53INP1* were the most significant up-regulated protein coding genes (Figure 1a). We identified also 317 DE lncRNAs of which 128 up-regulated and 189 down-regulated by ERα silencing. The most significant down-regulated lncRNAs were *RP11-68L18.1*, *MIR9-3HG*, and *LINC01016,* whereas *NKILA*, *AC144831.1*, and *LINC00657* were the most significant up-regulated lncRNAs (Figure 1b).

Functional enrichment analysis of biological processes for the DE genes evidenced mitotic cell cycle (GO:0000278), *mitotic nuclear division (GO:0007067)*, and *cell cycle phase transition (GO:0044770)* as the most enriched processes (Supplementary Table S2). Interestingly, different processes were enriched when up-regulated and down-regulated genes were considered separately. Specifically, siERα up-regulated genes were enriched for *neuron projection guidance (GO:0097485)*, *axon guidance (GO:0007411)*, and *epithelium development (GO:0060429)* while down-regulated genes were enriched for cell cycle- and DNA replication-related processes (Figure 1c).

To correlate apoERα genomic Binding Sites (aERBS) with gene regulation, we took advantage from our ERα ChIP-seq experiment performed in hormone-deprived MCF-7 cells [6]. As previously reported [6,21], analysis of apoERα binding mapped within 100 Kbp from a DE gene TSS revealed a clear bias in receptor binding close to down-regulated as compared to up-regulated genes (Supplementary Figure S1a). Specifically, 485 down-regulated genes (35.2%) compared to 300 up-regulated genes (27.0%) were associated to an apoERα Binding Site (aERBS).

To verify whether the apoERα binding involved in DE gene regulation occurs within SEs in MCF-7 cells, we have applied a bioinformatics strategy that is summarized in Supplementary Figure S1b. In detail, starting from H3K27ac ChIP-seq data of MCF-7 cultured in HD medium for 48 h and treated with “vehicle” as control for E2 treatment (GSE40129) [23], we predicted 438 SEs (Supplementary Table S3) characterized by a significant and wide H3K27ac enrichment as previously reported [24]. Then, we associated SE to the first proximal gene TSS and we ranked SEs based on the H3K27ac/input enrichment. Interestingly, among the top 50 SEs associated with the highest H3K27ac signal, we found pivotal luminal BC protein-coding genes, including *ESR1*, *GATA3*, and *FOXA1*. Furthermore, lncRNA genes were also associated with this SEs set, including *MALAT1* and *DSCAM-AS1*.

A subset of 227 SEs showed an overlap with aERBSs, hereafter referred to as “SE-aERBS”. Considering the DE genes mapped within 100 kbp from a SE-aERBS center, we associated 117 DE genes including protein coding genes like *ESR1*, *SPDEF*, *ZNF217*, and *RARA* (Supplementary Table S4). 35 lncRNA genes were also associated to an SE-aERBS and they were subdivided in the following biotypes: 17 antisense, 9 lincRNAs, three processed pseudogenes, two processed transcripts, two transcribed unprocessed pseudogenes, one snoRNA, and one lncRNA lacking experimental evidence but annotated in Gencode database (To be Experimentally Confirmed, TEC). Interestingly, 77% of DE genes associated to an aERBSs-SE were down-regulated upon apoERα silencing.

To understand whether SE-associated lncRNAs may represent a subset of the estrogen-responsive noncoding genes, we considered RNA-seq data from a time-course E2 experiment that is, an RNA-Seq experiment performed on MCF-7 cells collected after vehicle or E2 treatment (nine experimental time points: from 5 to 1280 min) (Supplementary Table S5; GSE62789) [25]. The heat map shown in Figure 1d represents the lncRNA log2FC differential expression upon apoERα silencing or during E2 treatment. This analysis uncovered 21 E2-responsive lncRNAs associated with an SE-aERBS. Specifically, eight lncRNAs down-regulated by E2 treatment were up-regulated by apoERα silencing; conversely, only three lncRNAs were up-regulated by E2 and down-regulated by siERα. Interestingly, the majority of lncRNA down-regulated by siERα were not significantly modulated by E2 treatment. Since the genes expressed at lower levels are biased toward high absolute log2FC, we verified the expression level of these lncRNAs in terms of Fragments Per Kilobase per Million (FPKM). As shown in Figure 1e, *DSCAM-AS1* was the lncRNA with the highest level of expression among our set of apoERα-regulated lncRNAs and its expression was not significantly affected by the E2 treatment as previously reported [21]. Among the lncRNA associated to a SE-aERBSs, we verified experimentally eight lncRNAs for their responsiveness to siERα or E2 treatment. As reported in Figure 1f, five out of eight lncRNAs showed a significant reduction of expression level in siERα-treated cells, while the lncRNA SRRM2-AS1 was significantly up-regulated. In E2-treated cells three lncRNAs were up-regulated while two tested lncRNAs were down-regulated. *DSCAM-AS1* was only slightly significant up-regulated after 24 h of E2 treatment (Figure 1g).

### 2.2. DSCAM-AS1 SE Characterization in Hormone-Deprived MCF-7

We focused our analysis on *DSCAM-AS1* in consideration of its unexpected abundance and unresponsiveness to E2, which suggest a peculiar hormone-independent transcriptional regulation involving SE regions. 

In the SE mapped upstream of the *DSCAM-AS1* locus, we observed nine different SE-aERBSs (named E1-9). To identify which of these regions is involved in *DSCAM-AS1* regulation, we used public ERα Chromatin Interaction Analysis with Paired-End-Tag sequencing (ChIA-PET) data of MCF-7 grown in full medium condition (GSE39495) [26]. We observed that *DSCAM-AS1* promoter interacts directly with two SE-aERBSs, E6 and E8, in particular E6 was characterized by the higher number of interactions (Supplementary Figure S1c). Overlap with aERBSs epigenetic classification performed in [27] confirmed that these regions are classified as active and transcribed enhancer (*EnhT*). Known long-range interactions were also confirmed using the *TFF1* gene as positive control for the analysis.

**Figure 2:**
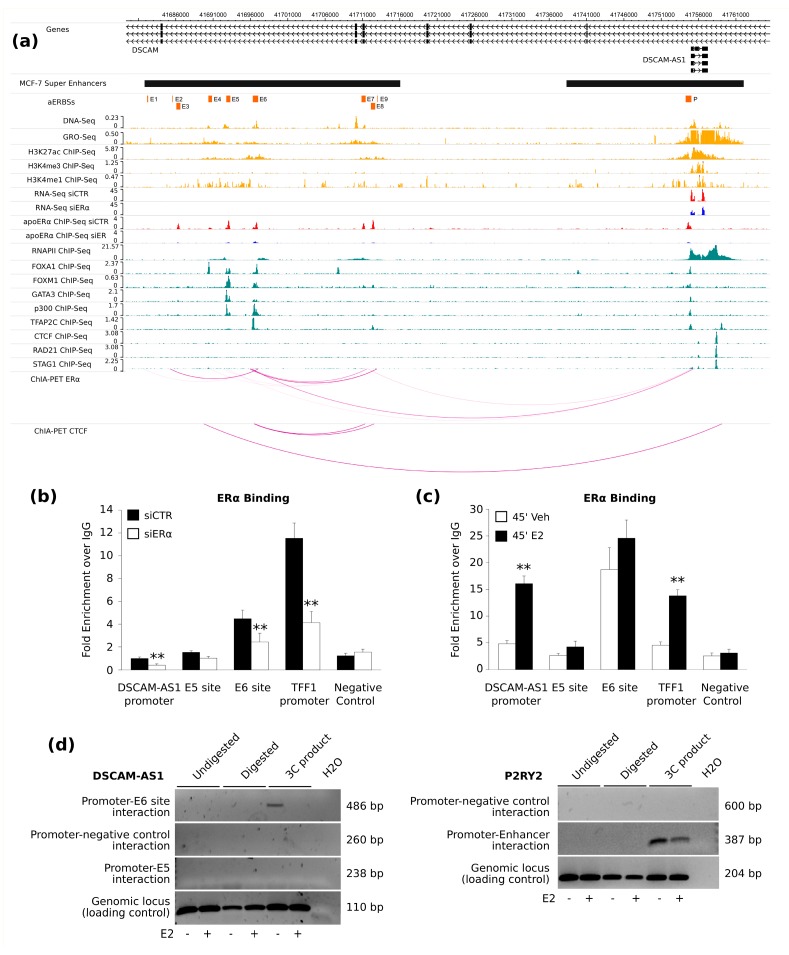
Characterization of *DSCAM-AS1* SE in MCF-7 cells. (**a**) WashU genome browser view of the *DSCAM-AS1* genomic locus, which is reported in association with apoERα ChIP-Seq and RNA-Seq reads enrichment in siCTR-(red) or siERα-(blue) transfected MCF-7 cells; and with GRO-Seq, ChIP-Seq profiles of histone modifications and DNase hypersensitivity signals (yellow), RNA-Pol II and eight TFs (teal-blue) obtained from vehicle treated MCF-7 cells (see the Materials and Methods section for detailed data source). ERα and CTCF ChIA-PET data performed in MCF-7 grown in full medium from ENCODE project are also included [26]. The coordinates of the predicted SE and aERBSs are reported at the top as black and orange boxes, respectively. ChIP-Seq genomic signal profiles are reported as read count per million sequenced reads. (**b**,**c**) Bar plots reporting ERα fold enrichment over IgG signal by ChIP-qPCR at *DSCAM-AS1* promoter and E5-E6-enhancers, *TFF1* promoter (positive control) and *KCNQ1OT1* promoter (negative control), in MCF-7 cells grown in HD medium transfected with siERα or siCTR (**b**); treated for 45′ with 17β-estradiol (E2) or vehicle (veh) (**c**). Standard error of four biological replicates; *p*-value by unpaired *t*-test: ** *p* < 0.01; (**d**) **Left.** Qualitative PCR reactions with primers detecting *DSCAM-AS1* promoter interaction with E5 or E6 enhancer sites; primers detecting *DSCAM-AS1* genomic region or non-interacting region; **Right**. Qualitative PCR reactions with primers detecting *P2RY2* promoter-enhancer interaction; primers detecting *P2RY2* genomic region or non-interacting region. All reactions were loaded on 2% agarose gel. Undigested and Digested samples represent DNA controls recovered during 3C libraries production.

Then, to explore the genomic and epigenomic context of the nine SE-aERBSs of the *DSCAM-AS1* locus, we took advantage of public MCF-7 ChIP-Seq data obtained in HD condition. Using the WashU genome browser, we integrated these data with the RNA-seq and ChIP-seq experiments of HD MCF-7 transfected with siERα or control siRNA (Figure 2a). As expected, the nine SE-aERBSs were characterized by an enhancer histone epigenetic pattern, with high DNaseI hypersensitivity, enhancer RNA transcription measured by Global Run-On and sequencing (GRO-Seq) [28], and H3K27ac and H3K4me1 enrichment. Conversely, the histone modifications at *DSCAM-AS1* TSS show typical promoter features, as H3K4me3 enrichment and high-level of RNA polymerase II (RNAPII) occupancy. In addition, the SE-aERBSs (particularly E5 and E6) were characterized by the enrichment of Forkhead Box A1 (FoxA1), Forkhead box protein M1 (FoxM1), histone acetyltransferase p300 and GATA Binding Protein 3 (GATA3), confirming the active status of these regulatory regions. Interestingly, the RNAPII ChIP-Seq signal at the *DSCAM-AS1* locus decreases rapidly in the region downstream *DSCAM-AS1*, where a CTCF binding sites is characterized by a ChIP-Seq enrichment of components of the cohesin complex RAD21 and STAG1 (GSE25021) [29]. To further confirm the candidate long-range chromatin interactions involving *DSCAM-AS1* locus and the SE-aERBSs, ERα and CTCF ChIA-PET data were visualized. This data confirms a consistent interaction between the *DSCAM-AS1* promoter and E5, E6, and E8 SE-aERBSs.

To investigate the role of apoERα in the regulation of *DSCAM-AS1* expression, we focused our analysis on E5 and E6 SE-aERBSs characterized by the highest apoERα signal and co-factor co-occupancy. By ChIP-qPCR we first evaluated ERα enrichment in MCF-7 cells maintained in HD medium and transfected with siERα or control siRNA. Knockdown of ERα was monitored by western blot, observing a reduced level of the protein (73.5%, Supplementary Figure S1d,e). The apoERα binding in E5, E6 and *DSCAM-AS1* promoter aERBSs was confirmed by ChIP-qPCR and it decreased significantly upon ERα silencing (Figure 2b), indicating that these are bona fide aERBS in the absence of hormones. The *TFF1* and *KCNQ1OT1* promoters were used as ERα-binding positive and negative controls, respectively. We also observed a decreasing trend of ERα binding at promoter and SE-aERBSs of three lncRNAs and a significant reduction of ERα binding at AC099850.1 and LINC00243 SEs upon ERα silencing (Supplementary Figure S1f), suggesting a general ERα-SE controlled regulation of lncRNAs expression in absence of estrogens.

Next, we wanted to ascertain if SE-aERBSs of DSCAM-AS1 were also estrogen-responsive. Thus, we evaluated ERα enrichment in MCF-7 cells grown in HD medium for four days and then treated with 17β-estradiol (E2) or vehicle for 45 min, by ChIP-qPCR. We observed a significant enrichment of ERα binding upon E2 treatment at the *TFF1* gene promoter used as a positive control, as well as on the *DSCAM-AS1* promoter [21] (Figure 2c). Interestingly, we noticed no significant enrichment of ERα signal upon E2 treatment at E5 and E6 SE-aERBSs regions suggesting an estrogen-independent ERα action on this region.

To verify the physical interaction between *DSCAM-AS1* promoter and SE, we performed a Chromosome Conformation Capture (3C) analysis. Interestingly, we detected E6-promoter interaction in vehicle-treated cells, whereas no interactions between the E5 site and *DSCAM-AS1* promoter were observed (Figure 2d). As a positive control, *P2RY2* promoter-enhancer interaction was confirmed. No signal was observed in either *DSCAM-AS1* or *P2RY2*-negative controls. This result gave us a first evidence of the direct interaction between ERα-bound enhancer and *DSCAM-AS1* promoter in absence of hormones.

**Figure 3:**
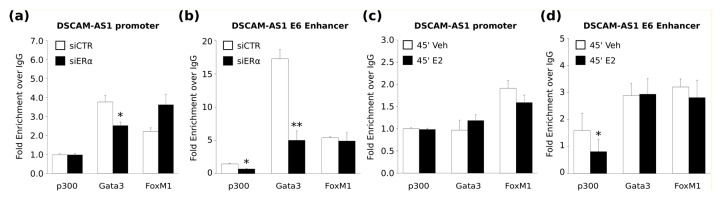
Analysis of ERα co-factor binding at *DSCAM-AS1* locus. (**a**,**b**) Bar plots reporting p300, GATA3 and FoxM1 fold enrichment over IgG signal at DSCAM-AS1 promoter (**a**) and E6-enhancer (**b**) by ChIP-qPCR in MCF-7 cells grown in HD and transfected with siCTR or siERα; (**c**,**d**) Bar plots reporting p300, GATA3 and FoxM1 fold enrichment over IgG signal at DSCAM-AS1 promoter (**c**) and E6-enhancer (**d**) by ChIP-qPCR in MCF-7 cells grown in HD and treated for 45′ with 17β-estradiol (E2) or vehicle (veh). Standard error of three biological replicates; *p*-value by unpaired *t*-test: * *p* < 0.05; ** *p* < 0.01.

To investigate if ERα knockdown also influences co-factors binding at the *DSCAM-AS1* enhancer region, we performed a ChIP-qPCR analysis against p300, Gata3 and FoxM1 proteins on MCF-7 cells maintained in HD and transfected with control or siERα. ERα silencing induced a significant decrement of GATA3 binding at both *DSCAM-AS1* promoter and E6-enhancer regions, while p300 binding was significantly downregulated only at E6 SE-aERBS (Figure 3a,b), suggesting that the activity of this SE critically depends on the presence of ERα. Since p300 and GATA3 protein levels did not change upon ERα silencing (Supplementary Figure S1d,e), we concluded that the observed reduced binding of these co-factors depends rather on the presence of ERα in the region than on a transcriptional regulation. On the contrary, FoxM1 binding was not significantly affected by ERα silencing.

Interestingly, we observed significant decrease of p300 occupancy within DSCAM-AS1 SE-aERBS upon E2 treatment (Figure 3c,d), suggesting a hormone-independent activation of *DSCAM-AS1* SE. By contrast, p300 binding on *DSCAM-AS1* promoter region showed no change upon E2 treatment.

Since CTCF binding sites were mapped downstream the *DSCAM-AS1* gene and a long-range interaction between this site and a site farther upstream to the SE was identified by ChIA-PET (Figure 2a), we asked whether CTCF binding could be involved in the *DSCAM-AS1* enhancer-promoter regulatory interactions. We studied CTCF binding downstream the *DSCAM-AS1* region and upstream the associated SE by ChIP-qPCR. Noteworthy, CTCF binding dropped significantly upon ERα-silencing in the upstream region of *DSCAM-AS1* SE (Supplementary Figure S1g), without any change of the CTCF protein level (Supplementary Figure S1d,e), strengthening again the central role of ERα in the maintenance of an active SE. Interestingly, we found that CTCF occupancy tends to decrease upstream the *DSCAM-AS1* SE and significantly diminishes downstream the *DSCAM-AS1* gene after E2 treatment, suggesting an hormone-independent CTCF insulation of *DSCAM-AS1* enhancer-promoter interaction. 

### 2.3. DSCAM-AS1 SE Characterization in Drug-Resistant BC Cell Lines

Since chromatin binding of luminal BC-specific TFs (ERα, Gata3, FoxM1) is enriched at *DSCAM-AS1*-SE, we verified whether the same genomic regions were predicted as an active SE in other BC cell lines. For this analysis, we used H3K27ac ChIP-Seq data from [30] in which the authors created different somatic cell fusions between luminal and basal BC cell lines. As reported in Supplementary Figure S2a the analysis of SEs in these cell lines revealed that *DSCAM-AS1* SE was predicted only in luminal MCF-7 and T-47D cell lines but not in any of the basal or basal/luminal fusion cell lines. The H3K27ac signal at SE is lower in T-47D cells, as compared to MCF-7, and almost absent in ZR-75.1 cells; surprisingly, we observed that DSCAM-AS1 expression is up-regulated by E2 in these cell lines (Supplementary Figure S2b,c).

**Figure 4:**
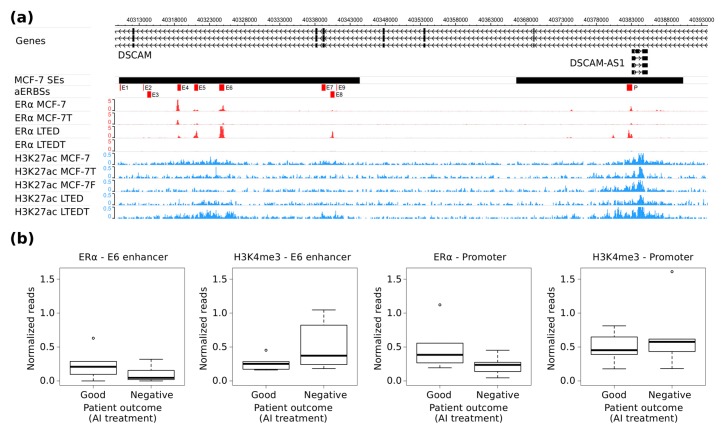
*DSCAM-AS1* SE characterization in drug-resistant BC cell lines. (**a**) WashU genome browser view of *DSCAM-AS1* genomic locus reporting at top the ChIP-Seq genomic signal profile of ERα and H3K27ac ChIP-Seq experiments performed in wild type MCF-7, MCF-7 resistant to Tamoxifen (MCF-7T), MCF-7 resistant to Fulvestrant (MCF-7F), Long Term Estrogen Deprived (LTED) MCF-7 cells, and LTED MCF-7 resistant to Tamoxifen (LTEDT) [31]. (**b**) Box plot reporting the normalized number of ERα and H3K4me3 ChIP-Seq reads counted at DSCAM-AS1 E6 enhancer (left) and promoter (right) considering data from patient responsive (Good prognosis, Good) or not (Negative prognosis, Negative) to Aromatase Inhibitors treatment (AI) [32]. Black dots represent outliers.

To further explore the relationship between *DSCAM-AS1* expression and the activity of the associated SE, we analyzed public ERα and H3K27ac ChIP-Seq data obtained in Long-Term Estrogen Deprived (LTED) models and Tamoxifen resistant model from [31]. As reported in Figure 4a, ERα binding at *DSCAM-AS1* SE occurs as well in wild-type as in Tamoxifen resistant and in LTED models. Only in a LTED model, which is resistant also to Tamoxifen, is the binding absent, despite the high H3K27ac ChIP-Seq signal still measured in this region. Noteworthy, the highest ERα ChIP-Seq signal at E6 SE-aERBS was observed in the LTED model.

Analysis of ERα and H3K4me3 ChIP-Seq data of primary tumor tissues from patients who respond or not to AI treatment [32] showed an higher H3K4me3 signal in AI-resistant than in AI-responder patients (Figure 4b); in particular, in four out of six cases of AI-resistant patients a clear H3K4me3 signal at *DSCAM-AS1* promoter and gene locus combines with a low but evident ERα ChIP-Seq signal at both E6 and *DSCAM-AS1* promoter (Figure 4b and Figure S2d).

Analysis of ERα and H3K4me3 ChIP-Seq data of primary tumor tissues from patients responding or not to AI treatment [32] showed higher H3K4me3 signal in AI-resistant than in AI-responder patients (Figure 4b); in particular, in four out of six cases of AI-resistant patients a clear H3K4me3 signal at *DSCAM-AS1* promoter and gene locus combines with a low but evident ERα ChIP-Seq signal at both E6 and *DSCAM-AS1* promoter (Supplementary Figure S2d).

## 3. Discussion

In this work, we have presented novel results that help understanding the role of Estrogen Receptor α in absence of hormones (apoERα), which is a critical issue for Breast Cancer (BC) patients given adjuvant treatment with Aromatase Inhibitors (AIs). We performed a deep triplicate RNA-Seq in hormone-depleted MCF-7 cells upon ERα silencing and, specifically, we were interested to understand whether Super Enhancers (SEs) were involved in sustaining the basal expression of a group of luminal-specific lncRNAs. Our study indicates that a set of apoERα dependent lncRNAs lie close to SEs, suggesting a strict dependence on these regions. Focusing on a representative lncRNA gene, *DSCAM-AS1*, we observed that aERBSs within the major SE in this region are active enhancers, also displaying long-distance contacts with the *DSCAM-AS1* promoter, even in the absence of hormones. Moreover, our data suggests that the chromatin domain confining this interaction may depend upon the estrogen-independent CTCF binding in proximity of *DSCAM-AS1*. Finally, comparison of SE activity in different BC cell lines revealed a relationship between ERα expression level and SE activity as well as *DSCAM-AS1* responsiveness to E2 treatment. Noteworthy, we confirmed the active epigenetic status of *DSCAM-AS1* locus in BC patients unresponsive to AI, but low ERα binding at SE was observed in these samples.

The identification of SEs helped us to understand the peculiar transcriptional program regulating cell-type specific gene expression. Recently, several evidences demonstrated that cell-specific enhancers play an important role in tumorigenesis [11]. Indeed, the study of SEs driving the expression of specific cancer associated lncRNAs may delineate an additional informative layer on cancer development.

One important limitation of our results is represented by the fact that the possible targets of SE were identified only based on the distance, i.e., we attributed to each SE the closest apoERα-regulated lncRNA gene. While on the basis of the reported frequency of enhancer-promoter interaction this is justified, the ENCODE project and other studies have reported that in a number of cases enhancers interact with promoters jumping over several genes [26]. Clearly, the matter would require direct long-range interaction studies using the Hi-C technology [33] and derivatives. Nevertheless, at least in the case of *DSCAM-AS1*, the prediction appeared correct and could be confirmed by our ChIA-PET network model.

The expression of the carcinoma-specific *DSCAM-AS1* is particularly interesting due to its abundance and close association with unliganded ERα activity. *DSCAM-AS1* loss impairs indeed BC cells proliferation and its expression is somehow implicated in Tamoxifen-resistance [21,22]. Here, we confirmed and extended previous results of our group [21] by validating ERα binding both in the promoter region and in putative enhancer elements that was very high in absence of estrogen, demonstrating only a slight increase after estradiol stimulation, and confirmed that this increase is not paralleled by transcriptional activation, whereas down-regulation of ERα by RNA interference led to significant transcriptional inhibition. On the contrary, low levels of the coding DSCAM transcript are detectable in breast cancer cells and no function of DSCAM in mammary tissues has been described to date. DSCAM mRNA slightly decreases upon ERα-silencing but it appears induced by estrogen [21,34], suggesting that the estrogen-induced re-localization of aERBSs might differentially regulate the expression of these sense and antisense transcripts in BC cells. Altogether, this data indicates a specific ligand-independent ERα program to sustain *DSCAM-AS1* expression in BC cells.

The presence of apoERα bound to an intronic element, which can increase quantitatively in response to estrogen but not transcriptionally, is not novel: a similar situation was reported for an intronic site in the retinoic acid receptor alpha 1 gene (*RARA*) [35] suggesting that such regulatory modality may be more frequent in lncRNA genes, but also present in protein-coding genes. Notably, also the *RARA* gene has a very important developmental function in epithelial mammary cells. One important novel finding in our data is represented by the state of activation of the aERBS within the major SE upstream the *DSCAM-AS1* gene. We evaluated p300 enrichment confirming the active status of these regulatory regions. In particular, the transcriptional coactivator p300 possesses intrinsic acetyltransferase activity and it was shown to play a critical role in transcriptional regulation [36]. Consistent with our results, p300 occupancy is a highly accurate method to identify enhancers and their associated activities [37,38]. Interestingly, we observed that ERα depletion induced a decrease of p300 occupancy, suggesting that the activity of these enhancers requires the presence of ERα. Moreover, p300 binding to the *DSCAM-AS1* enhancer is conserved rather in hormone-deprived medium than upon estrogen treatment, indicating that this SE is not an early estrogen target site. However, the dynamic distribution of p300 in BC cells in the presence or absence of estrogenic hormones and its effect on the prognosis of BC are poorly understood [38].

Our analysis confirms the model where apoERα collaborates with other TFs to maintain the luminal epithelial enhancer landscape [6]. These cooperation are underlined by the cross-regulatory loop involving GATA-3 and ERα or AP2γ and ERα, which sustain each other’s expression in BC cells [39,40]. Interestingly, we also observed that ERα depletion induced a decrease of GATA3 occupancy; therefore, the regulation of *DSCAM-AS1* gene expression seems to be based on long range interaction between apoERα-bound enhancer and the gene promoter as previously described for some estrogen-dependent ERα target genes such as *TFF1* and *GREB1* [41]. Noteworthy, our analysis suggests that only the E6 aERBS is involved in *DSCAM-AS1* regulation. In fact, both ERα binding and 3C analysis show very low activity of E5, also reflecting public ChIA-PET data of MCF-7 cells grown in full medium, i.e., under low hormone concentrations [26].

Therefore, we attempted *DSCAM-AS1* promoter-enhancer interaction before and after estrogen stimulation and we could detect at least the E6-promoter interaction, further demonstrating that this enhancer is active even in the absence of estrogen. However, it should be emphasized that this is a preliminary and qualitative result, which should be confirmed by further experiments to quantify the frequency of *DSCAM-AS1* promoter-enhancer interaction in different experimental conditions. This data will allow understanding if this interaction is more favored or stabilized in absence of hormones, as we hypothesize.

The eukaryotic genome is organized into functionally and structurally distinct chromatin domains which orchestrate spatial and temporal gene regulation. CTCF, the most characterized insulator-binding protein, is directly involved in chromatin architecture by the formation of barriers which limit the diffusion of chromatin states and promote tissue-specific local chromatin hubs. CTCF binding to insulator sequences can partition the genome into distinct ERα-regulatory blocks [42] defining sensitive and insensitive regions [43]. However, redistribution of CTCF binding sites after E2 treatment was not generally observed [44]. Since CTCF binding sites were mapped around *DSCAM-AS1* and a long-range interaction was observed between a site close to *DSCAM-AS1* 3’ end and a site farther upstream the SE, we asked whether CTCF binding could justify the peculiar mode of action of the *DSCAM-AS1* enhancer/promoter regulatory system. Noteworthy, we found that CTCF occupancy upstream *DSCAM-AS1* decreases after ERα silencing in HD medium, whereas it decreases significantly downstream the *DSCAM-AS1* gene region after estradiol stimulation, suggesting that estrogen, or its absence, may induce a redistribution of long-range interactions. Taken together, these results suggest a non-trivial regulatory model at this site, which may involve channeling SE action on the *DSCAM-AS1* promoter in cancer cells overexpressing ERα due to strict domain definition by CTCF. We also supposed that the reduction of CTCF signal upon estradiol stimulation indicate that these enhancers might regulate distal genes surmounting the protein insulator. Interestingly, the analysis of public RNA-Seq data MCF-7 transfected with siCTCF and treated with E2 for 3 h show a reduction of *DSCAM-AS1* level in siCTCF-treated cells (Supplementary Figure S2e).

Our data suggest a model in which CTCF protein acts by insulating the interaction between *DSCAM-AS1* and associated SE. Within the SE a hierarchical ERα binding starting from E6 aERBS maintains the long-range chromatin contact even in hormone-deprived cells. A similar hierarchical model was recently proposed by different groups [14,45], however, if this model can apply to the regulation of other ERα-dependent lncRNAs is not known at present and will require extension of these studies in the near future.

Finally, analysis of the *DSCAM-AS1* locus in several BC cell lines suggested a direct correlation between the level of ERα expression and the *DSCAM-AS1* SE activation status. Indeed, in MCF-7, (the BC cell line characterized by the highest ERα expression), the *DSCAM-AS1* SE is completely active, while only the *DSCAM-AS1* promoter region is active in ZR-75.1 (characterized by a lower ERα expression). Furthermore, the LTED cell model system, which is characterized by an elevated ERα expression [46], were also characterized by the highest ERα SE occupancy among all the cell lines analyzed. However, given the extensive genetic and epigenetic heterogeneity driving the acquisition of a hormone-independent phenotype in the different LTED models [31], limited general conclusion can be translated to the clinical settings.

Analysis of ERα and H3K4me3 ChIP-Seq data clearly show extensive heterogeneity among patients. Using these data, we were not able to confirm a strong ERα binding at *DSCAM-AS1* SE, but we clearly observed that *DSCAM-AS1* locus is active in four out six patients non-responsive to AI treatments. Since patients with a good prognosis present no H3K4me3 signal at DSCAM-AS-SE, its activated-status is correlated with a negative prognosis, suggesting it as epigenetic-predictor of AI-treatment response. As far as the other common endocrine treatment is concerned, i.e., Tamoxifen, Niknafs and coworkers reported that Tamoxifen-resistant patients display ERα binding to *DSCAM-AS1* promoter, as opposed to Tamoxifen responders [22]. Using the same data [47], we additionally examined ERα binding at the *DSCAM-AS1* SE, confirming that ERα binding is limited to non-responder patients (Supplementary Figure S2d,f). Altogether, these data support the fact that *DSCAM-AS1* does not behave as a typical estrogen-responsive gene, since its active transcriptional status is maintained both in absence of estrogen (AI) and in the presence of antagonistic ERα ligands (Tamoxifen).

Moreover, *DSCAM-AS1* genes was included in the gene classifier of drug responsiveness proposed by Jansen and colleagues [32].

In our lab, we are actively exploring *DSCAM-AS1* expression in tissues of BC patients to demonstrate it possible role as hormone-responsiveness marker. At the same time, several other lncRNA loci are being studied to understand unliganded ERα action in luminal breast cancer.

## 4. Materials and Methods

### 4.1. Cells Culture and Treatment

MCF-7 and ZR-75.1 cells were routinely grown in DMEM medium supplemented with 10% heat-inactivated FBS, 2 mM l-glutamine, 50 U/mL penicillin and 50 μg/mL streptomycin. T-47D cells were routinely grown in RPMI medium supplemented with 10% heat-inactivated FBS, 2 mM l-glutamine, 50 U/mL penicillin and 50 μg/mL streptomycin. Hormone-deprived medium (HD) was obtained from phenol red-free DMEM supplemented with 5% charcoal-dextran-treated serum. MCF-7 cells were grown at least 4 days in HD medium before 17β-estradiol treatment (E2). 17β-estradiol (Sigma -Aldrich, St. Louis, MO, USA) was added at a final concentration of 10–8 M.

### 4.2. Small Interfering RNA (siRNA)

MCF-7 cells were grown in HD medium (for two days in this conditioned medium) before being transfected with siRNAs using Lipofectamine2000 (Life Technologies, Carlsbad, CA, USA), according to the reverse transfection protocol. Stealth RNAi™ siRNAs from Invitrogen were used to target ERα mRNA (ESR1HSS103376, ESR1HSS103377, ESR1HSS176619); stealth RNAi™ siRNA Negative Control medium GC was used as a control (siCTR). Any experiment was carried out 48 h after siRNA transfection in order to achieve the maximum efficiency of RNA interference.

### 4.3. RNA-Seq

MCF-7 cells were grown for 2 days in HD medium before siRNAs transfection (siCTR or siERa). RNA was harvested after 48 h from transfection by RNA isolation kit (RNeasy Mini Kit, Qiagen 74104). RNA quality check (RNA integrity number (RIN) > 8) was achieved with Fragment Analyzer (Advanced Analytical Technologies, Inc, Ankeny, IA, USA) and quantified with Qubit (Qubit™ RNA HS Assay Kit, ThermoFisher Scientific, Waltham, MA, USA;Q32852). Two μg of RNA were polyA+ selected and RNA-Seq libraries were constructed using Illumina TruSeq RNA sample prep kit (TruSeq™ RNA Sample Prep Kit v2-Set B, Illumina, RS-122-2002). Paired-end (PE) cluster generation was performed using cBot on Flow Cell v3 (TruSeq PE Cluster Kit v3-cBot-HS, Illumina, PE-401-3001). Sequencing of libraries was performed on the HiSeq sequencing system (Illumina, San Diego, CA, USA).

Raw and processed RNA-Seq data were deposited at GSE108693.

### 4.4. RNA-Seq Data Analysis

Raw RNA-Seq reads were aligned using *TopHat* v2 [48]. Gencode v24 was used as reference set of gene annotations. Read count was performed using *featureCounts* algorithm [49] and read count tables were normalized with *DESeq2* package [50]. Normalized read counts were converted to fragments per kilobase of exons per million fragments mapped (FPKM) considering the length of the longest isoform of each gene and the millions of reads counted by featureCounts.

Differentially expression between siCTR and siERα-treated MCF-7 was performed using *DESeq2* in default settings. A gene was considered as differentially expressed if associated with an adjusted *p*-value < 0.001. To identify genes differentially expressed in the time-course E2-treatment experiment, *maSigPro* R package [51] was applied in default setting by considering the adjusted *p*-value computed using the p.vector function of the package. A gene was considered as differentially expressed if associated with an adjusted *p*-value < 0.05. Gene to SEs association was performed by computing the distance between gene TSSs and each SE center. Only genes mapped within 100 kbp from a SE region were considered for the analysis.

Gene set functional analysis was performed using Enrichr algorithm [52].

Analysis of GRO-Seq datasets from GSE45822 was performed using Bowtie v2.1.0 [53] in default settings and the *local* option.

### 4.5. Super Enhancer Prediction

A H3K27ac ChIP-Seq dataset performed on 48-h hormone-deprived MCF-7 cells treated with vehicle (GSM986079) was used for the identification of SE regions. Raw ChIP-Seq reads were aligned using Bowtie 2.0 algorithm in default settings [53]. Significant H3K27ac ChIP-Seq peaks were called with MACS 2.1.0 [54]. The input ChIP-Seq experiment was used as background for the analysis (GSM986091). These peaks were used as input for the ROSE algorithm [24] analysis applied in default settings. Only SEs overlapped against apoERα ChIP-Seq peaks defined in [6] were selected for the analysis. The set of SEs predicted in other BC cell lines were retrieved from [30] supplemental material. These SE coordinates were converted in hg38 using LiftOver algorithm (https://genome.ucsc.edu/cgi-bin/hgLiftOver).

### 4.6. Long Range Chromatin Interaction Network

The network model of Gene-aERBSs chromatin interaction was generated using the ChIA-PET data from GSE39495. Each pair of interacting genomic regions was overlapped with the aERBSs and gene promoter coordinates. A promoter was defined as a region of −2 kbp and +500 bp around the gene TSS. Each aERBS was equally extended by 1 kbp in each size. The average number of interacting region between each aERBS-gene, gene-gene, or aERBS-aERBS pair was computed and represented as link width between the connected nodes. Gene were color-coded according to their expression log2FC in the siERα RNA-Seq experiment while aERBS nodes were colored according to their epigenetic classification defined in [27].

### 4.7. DSCAM-AS1 Locus Analysis

The analysis of *DSCAM-AS1* locus and upstream enhancer regions was performed using the Washu Genome Browser [55]. ChIP-Seq genomic signals from public experiments performed in hormone-deprived MCF-7 were defined by realignment of raw ChIP-Seq reads using Bowtie 2.0 algorithm in default settings. Then, alignment bam files were converted to bedgraph files using BEDTools algorithm [56]. Each ChIP-Seq genomic signal was converted in count per millions by dividing the read coverage data by the million number of sequencing reads in each experiment. In the analysis data from a ChIP-Seq performed against H3K27ac (GSM1115993), H3K4me3 (GSM1382470), H3K4me1 (GSM1382469), RNAPII (GSM1116656), FoxA1 (GSM588929), FoxM1 (GSM1001003), Gata3 (GSM986074), p300 (ERR045733), AP2γ (GSM588927), CTCF (GSM822308), Rad21 (GSM614613), and Stag1 (GSM614616) were considered. ERα ChIP-Seq and RNA-Seq data from siCTR or siERα-transfected MCF-7 (GSE53533) were also considered in the analysis. DNase-seq were obtained from GSM1024764. ERα and H3K4me3 ChIP-Seq data from AI-treated patients were obtained from GSE40867 while data from Tamoxifen-treated patients were retrieved from GSE32222.

Data from ENCODE long-range chromatin interaction experiments were analyzed by considering the ERα and CTCF ChIA-PET experiments performed in complete-medium grown MCF-7. The processed data of these experiments were provided by the public track hub “long-range chromatin interaction experiments” of the Washu Genome Browser.

### 4.8. Protein EXTract and Western Blot Analysis

Whole-cell lysate was harvested from organic-phenol phase after RNA purification in Purezol™ reagent (Bio-Rad, Hercules, CA, USA). 70 μg of protein extracts were denatured by boiling in 6x Loading Dye, fractionated by SDS-PAGE electrophoresis in 6% polyacrylamide gel and finally transferred on PVDF membrane (Millipore). Antibodies used were designed against ERα (Santa Cruz Biotechnology, Dallas, TX, USA; sc543 and sc-7207), CTCF (Merck-Millipore, Burlington, MA, USA; 07-729), GATA3 (Santa Cruz Biotechnology, Dallas, TX, USA; sc-268), p300 (Santa Cruz Biotechnology Dallas, TX, USA sc-585), FOXM1 (Santa Cruz Biotechnology Dallas, TX, USA sc-376471) and Hsp90 (Cell Signaling Technology, Danvers, MA, USA; 4874) proteins. Proteins quantitation was performed with Volume Analysis Tool from Quantity One™ software, version 4.6.6 (Bio-Rad).

### 4.9. RNA Isolation and Quantitative Real-Time PCR (qRT-PCR) 

RNA was isolated from MCF-7 cells using Purezol™ reagent (Bio-Rad). All total isolated RNAs were subjected to DNase treatment to remove contaminating genomic DNA (ezDNase™ Enzyme, Invitrogen, Carlsbad, CA, USA; 11766051). First strand cDNA synthesis from 250 ng of total RNA template was performed with SensiFAST cDNA Synthesis Kit (Bioline, London, UK; BIO-65054), followed by SYBR-green qRT-PCR amplification (iTaq UniverSYBR Green, Bio-Rad 1725124). Real-time PCR primers for human 18S, ERα, GREB1 RNAs were purchased from Qiagen (Hilden, Germany; QuantiTect™ Primer Assay). Custom expression-primer pairs are reported in Supplemental Table S6.

### 4.10. Chromatin Immunoprecipitation Assay (ChIP)

MCF-7 cells were grown for 2 days in HD medium before siRNAs transfection or 45 min of E2 treatment. ChIP experiments were then performed as follows: cells were cross-linked by addition of formaldehyde at 1% final concentration (Formaldehyde solution, Sigma-Aldrich 252549) and incubated 10 min at 37 °C. Cross-linking was stopped by addition of glycine solution to a final concentration of 0.125 M for 5 min on a shaker at room temperature. Cross-linked cells were then washed twice with ice-cold PBS supplemented with complete protease inhibitors cocktail and collect by scraping (Protease Inhibitor Cocktail, Sigma-Aldrich P2714-1). Cell pellets were subjected to lysis on ice for 10 min with Lysis Buffer 1 (5 mM Pipes pH 8, 85 mM KCl, 0.5 % NP40) supplemented with complete protease inhibitors cocktail. Subsequently, nuclei pellets, obtained by a 5 min spin cycle at 4 °C (4000 rpm) were exposed once again to 10 min lysis in Lysis Buffer 2 (1% SDS, 5 mM EDTA, 50 mM Tris-HCl pH 8.1) supplemented with complete protease inhibitors cocktail. Total extracted chromatin was sonicated to an average size of 250–500 bp by using an immersion sonicator device. The desired fragments size was checked on 1.2% agarose-gel and quantified by Nanodrop spectrophotometer at 280 nm, in order to use 50 μg of chromatin per IP. 10 μL (1%) of chromatin extracts was recovered as input normalization-control for each experimental condition. Chromatin extracts were diluted with IP-buffer (1% Triton X-100, 2 mM EDTA, 20 mM Tris-HCl of pH 8.1, 150 mM NaCl, supplemented with complete protease inhibitors) and incubated overnight with the specific antibody or IgG at 4° C on a rotating platform. Protein A and G sepharose-beads (GE Healthcare Life Sciences; Little Chalfont, UK; 17-5280-01 and 17-0618-01 respectively) were pre-coated with IP buffer supplemented with 5% BSA, in order to reduce nonspecific antibody binding. Upon 2 h of Protein A or G sepharose-beads incubation (depending on antibody source, i.e., rabbit or mouse respectively), samples were washed sequentially for 5 min, on a rotating platform with 1 mL of three different Washing Buffer (Washing buffer 1: 0.1% SDS, 1% Triton X-100, 2 mM EDTA, 20 mM Tris HCl pH 8, 150 mM NaCl; Washing buffer 2 : 0.1 SDS, 1% Triton X-100, 2 mM EDTA, 20 mM Tris HCl pH 8, 500 mM NaCl; Washing buffer 3: 0.25 M LiCl, 1% NP40, 1% Na DOC, 1 mM EDTA, 10 mM Tris HCl pH8) and twice with TE buffer (10 mM Tris HCl pH8, 1 mM EDTA). After complexes elution at RT with elution buffer (1% SDS, 0.1 M NaHCO3), DNA fragments were de-crosslinked at 65 °C overnight with NaCl 5 M and by 1 h of proteinase K treatment. DNA purification was achieved with Phenol:Chloroform:IAA (25:24:1 UltraPure™ formulation, Ambion AM9730) according to the manufacturer’s instructions. Quantitative Real-time PCR was carried out on ChIP-enriched DNA using SYBR Green Master Mix. ChIP enrichment was normalized on input samples (1% of total chromatin used per IP) and expressed as fold-enrichment of specific binding over the control nonspecific IgG binding. Antibodies against human ERα (Santa Cruz Biotechnology, Dallas, TX, USA; sc534X, sc7207X), p300 (Santa Cruz Biotechnology, Dallas, TX, USA; sc-585X), CTCF (Merk-Millipore, Burlington, MA, USA; 07-729), GATA3 (Santa Cruz Biotechnology, Dallas, TX, USA; sc-268X), FOXM1 (Santa Cruz Biotechnology, Dallas, TX, USA; sc-376471X) and normal rabbit IgG (Merk-Millipore, Burlington, MA, USA; 12-370) were used in this assay. Custom ChIP primer pairs are reported in Supplementary Table S6.

### 4.11. Qualitative Chromosome Conformation Capture (3C-PCR)

MCF-7 cells were grown for 2 days in HD medium before 45 min of E2 treatment. 3C procedure was performed essentially as described by [8] with some modifications: Step: Crosslinking reaction. As well as for ChIP experiments, cells were cross-linked by addition of formaldehyde (1% final concentration) and incubated 10 min at 37 °C. Cross-linking was stopped by addition of glycine solution to a final concentration of 0.125 M for 5 min on a shaker at room temperature. Cross-linked cells were then washed twice with ice-cold PBS supplemented with complete protease inhibitors cocktail and collect by scraping. Cell pellets were subjected to lysis on ice for 10 min with ChIP Lysis Buffer 1 supplemented with complete protease inhibitors cocktail. Subsequently, nuclei pellets, obtained by a 5 min spin cycle at 4 °C (4000 rpm) were resuspended with 100 μL of 3C buffer (50 mM Tris-HCl pH 8.0; 50 mM NaCl; 10 mM MgCl2; 1 mM DTT). Step 2: Permeabilization and digestion. SDS (final concentration 0.2%) was added and samples were incubated at 37 °C for 1 h while shaking at 300 rpm. Then, Triton X-100, diluted in 1X ligase buffer (NEB, B0202S), was added at 1.2% final concentration and samples were incubated at 37 °C for 1 h while shaking at 300 rpm. A 10 μL aliquot was taken by pipetting on the wall of the tube (without mixing before). This sample corresponds to undigested DNA. DNA digestion will be done by adding a total of 450 U of restriction enzyme (BglII and NsiI, NEB R0144L and R0127L respectively) overnight at 37 °C while shaking at 300 rpm. A 4.5 μL aliquot was taken by pipetting on the wall of the tube (without mixing before). This sample corresponds to digest DNA. Step 3: Restriction enzyme inactivation and ligation of chimerical products. SDS (final concentration 0.2%) was added and samples were incubated at 37 °C for 30 min while shaking at 300 rpm. Samples were then diluted 4 times in 1X ligase buffer and Triton X-100, diluted in 1X ligase buffer, was added at 1% final concentration. Finally, samples were incubated at 37 °C for 2 h while shaking at 200 rpm. After a centrifugation of 1 min/2200 g/4 °C, 3.27 mL of supernatant were taken off such as 500 μL remain in the tube. On ice, 195 U of ligase (T4 DNA ligase, NEB M0202L) were carefully mixed and re-suspended with the pellet, then incubated overnight at 16 °C. After incubation this sample corresponds to 3C library. Step 4: Reversal of crosslinking. 3C libraries were 7 times diluted in 2X Proteinase K buffer (5 mM EDTA pH 8.0; 10 mM Tris-HCl pH 8.0; 0.5% SDS) and water. Undigested and digested samples were supplemented with 500 μL of 1xPK buffer. 100 μg or 20 μg of proteinase K (20 mg/mL) were mixed to the 3C libraries or to the undigested and digested DNA, respectively. All samples were incubated during 1 h at 50 °C and then during 4 h at 65 °C to reverse the crosslinking reaction. Step 5: DNA purification. 1 volume of Phenol:Chloroform:IAA was added to each sample (i.e., 4 mL to 3C library and 500 μL to the undigested and digested samples) and vigorously mixed. Following a centrifugation 10 min/16,100 g/RT, the supernatant aqueous phase was recovered in a new tube. 2 volumes of absolute ethanol and NaCl to a final concentration of 250 mM were added to each sample, mixed and let at −20° overnight to precipitate DNA. Following a centrifugation 20 min/16,100 g/4 °C, pellets were washed with 70% ethanol and finally re-suspended in water (150 μL for 3C libraries; 60 μL for the undigested and digested samples). Step 6: Qualitative PCR. After quantification at Nanodrop spectrophotometer, qualitative PCR (HiFi-Taq polymerase™ kit, Life Technologies, 11304-011) was carried out on 50 ng DNA of each samples by using the following primer pairs: Promoter-Gene, to detect genomic DNA (loading control); Promoter-Enhancer site, to detect the predicted interaction (3C products); Promoter-Enhancer nearby genomic region, as a negative control (non-interacting regions). Validated primers on *P2RY2* gene-enhancer interaction were used as a technique positive control [41] and custom primers were used to study *DSCAM-AS1* promoter-enhancer interaction. Primers are reported in Supplemental Table S6. PCR products were checked on 2% agarose-gel.

## Supplementary Materials

Supplementary materials can be found at http://www.mdpi.com/1422-0067/19/2/593/s1.

## Abbreviations

SE	Super Enhancer
TSS	Transcription Start Site
ERα	Estrogen Receptor α
aERBS	ApoER α Binding Site
SE-aERBS	ApoER α Binding Site associated to a Super Enhancer region
3C	Chromatin Conformation Capture
ChIA-PET	Chromatin Interaction Analysis with Paired-End-Tag sequencing
GRO-Seq	Global Run-On Sequencing
HD	Hormone Deprived Medium
E2	17β-estradiol
DE	Differentially Expressed
LTED	Long-Term Estrogen Deprived cells
AIs	Aromatase Inhibitors
