# Supplementary material for: Luminal lncRNAs Regulation by ERα-Controlled Enhancers in a Ligand-Independent Manner in Breast Cancer Cells
Source: Int J Mol Sci. 2018 Feb 16;19(2):593. doi: 10.3390/ijms19020593 (PMC5855815; doi:10.3390/ijms19020593)
Supplement: Supplementary file 1 [file ijms-19-00593-s001.zip › ijms-262598-Supplementary Update/Supplementary_Figures.pdf]

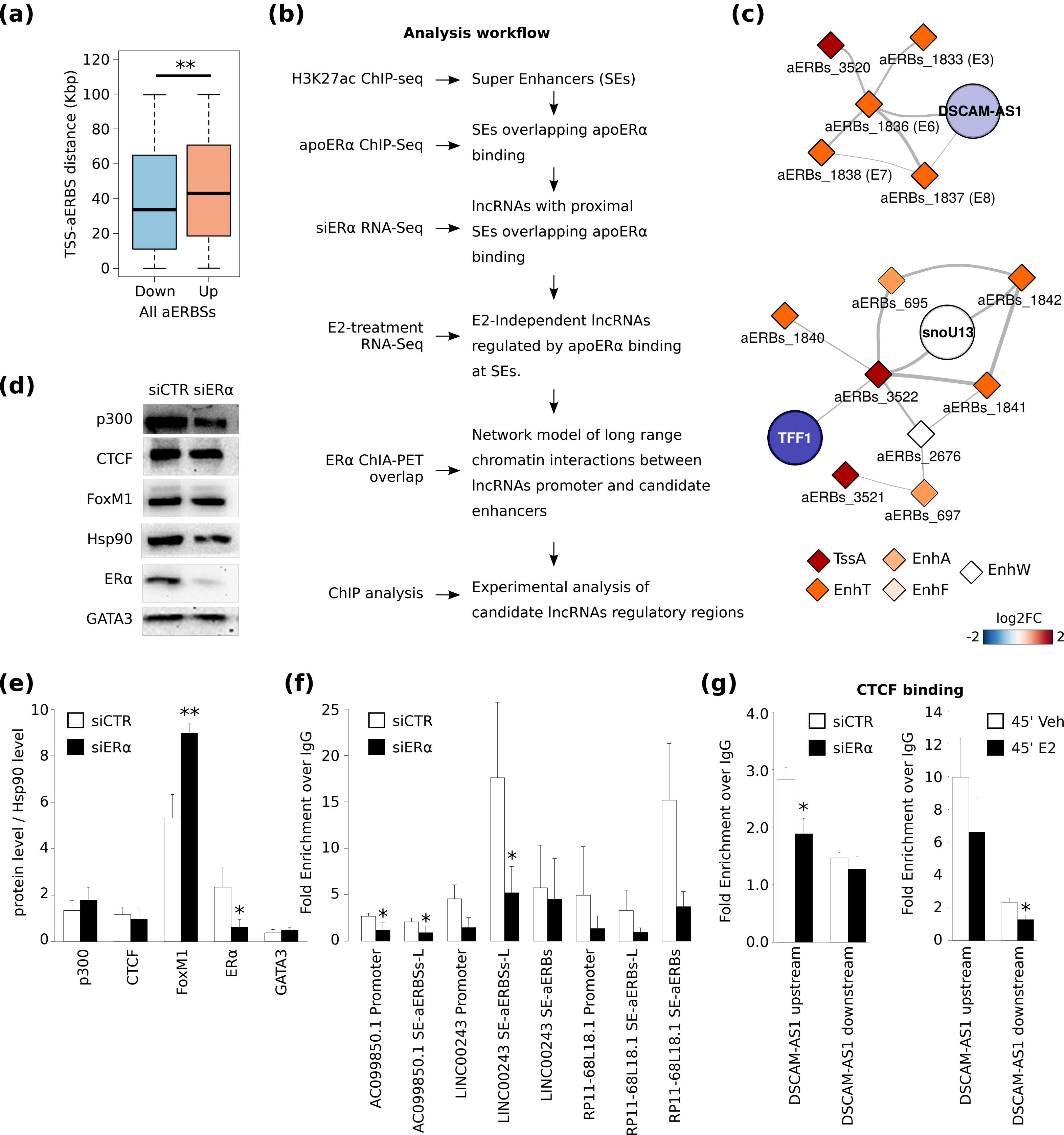

(a)
TSS-aERBS distance (Kbp)
**
Down
Up
All aERBSs
(b)
Analysis workflow
H3K27ac ChIP-seq
Super Enhancers (SEs)
apoERα ChIP-Seq
SEs overlapping apoERα binding
siERα RNA-Seq
lncRNAs with proximal SEs overlapping apoERα binding
E2-treatment RNA-Seq
E2-Independent lncRNAs regulated by apoERα binding at SEs.
ERα ChIA-PET overlap
Network model of long range chromatin interactions between lncRNAs promoter and candidate enhancers
ChIP analysis
Experimental analysis of candidate lncRNAs regulatory regions
(c)
aERBs_3520
aERBs_1833 (E3)
DSCAM-AS1
aERBs_1836 (E6)
aERBs_1838 (E7)
aERBs_1837 (E8)
aERBs_695
aERBs_1842
aERBs_1840
snoU13
aERBs_3522
aERBs_1841
TFF1
aERBs_2676
aERBs_3521
aERBs_697
TssA
EnhA
EnhW
EnhT
EnhF
log2FC
-2
2
(d)
siCTR siERα
p300
CTCF
FoxM1
Hsp90
ERα
GATA3
(e)
protein level / Hsp90 level
siCTR
siERα
p300
CTCF
FoxM1
ERα
GATA3
(f)
Fold Enrichment over IgG
siCTR
siERα
AC099850.1 Promoter
AC099850.1 SE-aERBSs-L
LINC00243 Promoter
LINC00243 SE-aERBSs-L
LINC00243 SE-aERBs
RP11-68L18.1 Promoter
RP11-68L18.1 SE-aERBs-L
RP11-68L18.1 SE-aERBs
(g)
CTCF binding
Fold Enrichment over IgG
siCTR
siERα
45' Veh
45' E2
DSCAM-AS1 upstream
DSCAM-AS1 downstream

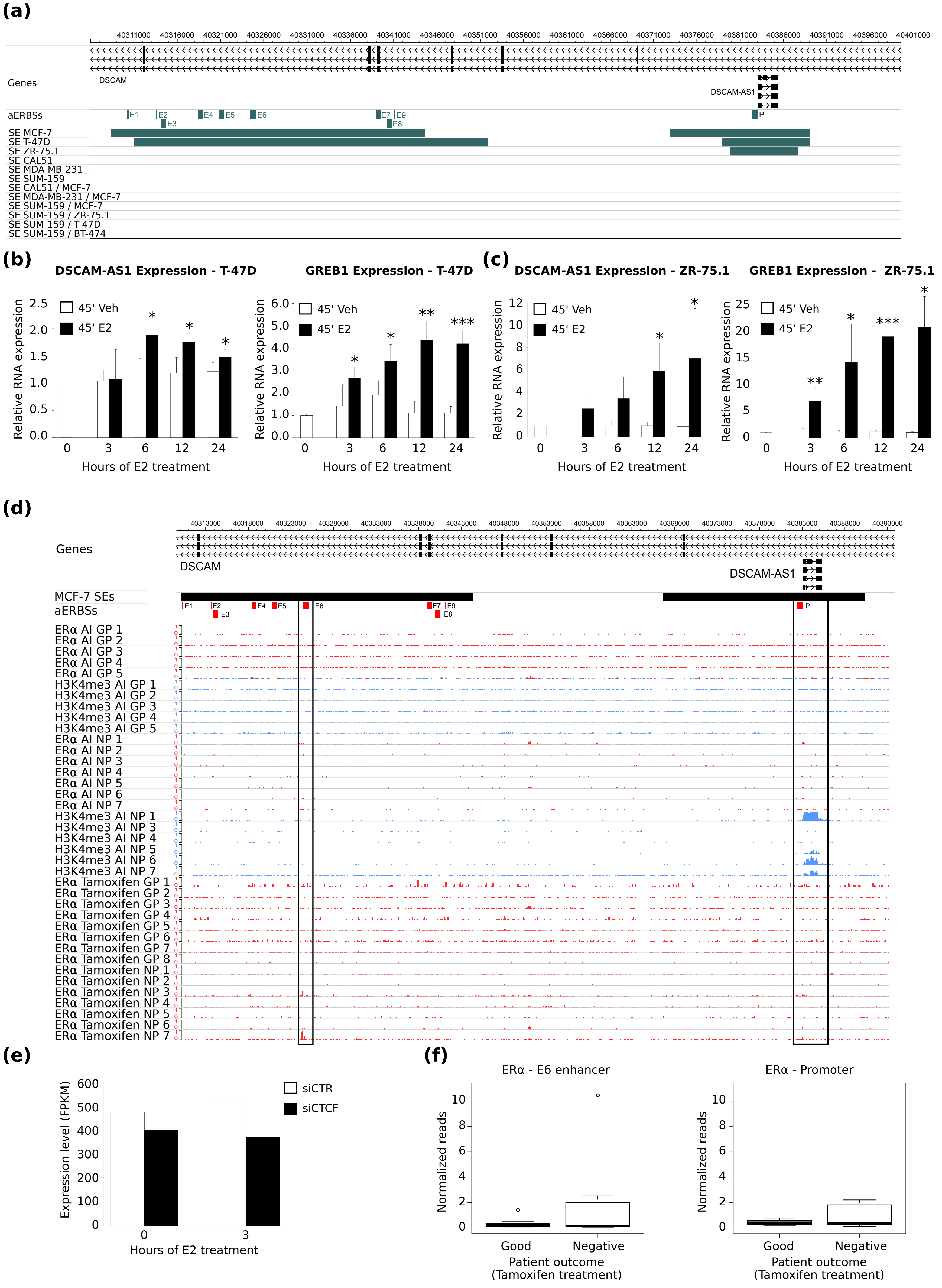

(a)
40311000 40316000 40321000 40326000 40331000 40336000 40341000 40346000 40351000 40356000 40361000 40366000 40371000 40376000 40381000 40386000 40391000 40396000 40401000
Genes
DSCAM
DSCAM-AS1
aERBSs
E1 E2 E3 E4 E5 E6 E7 E9 E8 P
SE MCF-7
SE T-47D
SE ZR-75.1
SE CAL51
SE MDA-MB-231
SE SUM-159
SE CAL51 / MCF-7
SE MDA-MB-231 / MCF-7
SE SUM-159 / MCF-7
SE SUM-159 / ZR-75.1
SE SUM-159 / T-47D
SE SUM-159 / BT-474
(b)
DSCAM-AS1 Expression - T-47D
GREB1 Expression - T-47D
(c)
DSCAM-AS1 Expression - ZR-75.1
GREB1 Expression - ZR-75.1
45' Veh
45' E2
Relative RNA expression
Hours of E2 treatment
0 3 6 12 24
(d)
Genes
DSCAM
DSCAM-AS1
MCF-7 SEs
aERBSs
E1 E2 E3 E4 E5 E6 E7 E9 E8 P
40313000 40318000 40323000 40328000 40333000 40338000 40343000 40348000 40353000 40358000 40363000 40368000 40373000 40378000 40383000 40388000 40393000
ERα AI GP 1
ERα AI GP 2
ERα AI GP 3
ERα AI GP 4
ERα AI GP 5
H3K4me3 AI GP 1
H3K4me3 AI GP 2
H3K4me3 AI GP 3
H3K4me3 AI GP 4
H3K4me3 AI GP 5
ERα AI NP 1
ERα AI NP 2
ERα AI NP 3
ERα AI NP 4
ERα AI NP 5
ERα AI NP 6
ERα AI NP 7
H3K4me3 AI NP 1
H3K4me3 AI NP 3
H3K4me3 AI NP 4
H3K4me3 AI NP 5
H3K4me3 AI NP 6
H3K4me3 AI NP 7
ERα Tamoxifen GP 1
ERα Tamoxifen GP 2
ERα Tamoxifen GP 3
ERα Tamoxifen GP 4
ERα Tamoxifen GP 5
ERα Tamoxifen GP 6
ERα Tamoxifen GP 7
ERα Tamoxifen GP 8
ERα Tamoxifen NP 1
ERα Tamoxifen NP 2
ERα Tamoxifen NP 3
ERα Tamoxifen NP 4
ERα Tamoxifen NP 5
ERα Tamoxifen NP 6
ERα Tamoxifen NP 7
(e)
siCTR
siCTCF
Expression level (FPKM)
Hours of E2 treatment
0 3
(f)
ERα - E6 enhancer
ERα - Promoter
Normalized reads
Good
Negative
Patient outcome
(Tamoxifen treatment)

Legends to Supplementary Figures

Supplementary Figure 1. Identification and characterization of SE-aERBSs. (**a**) Box plot reporting the distance between siERα up-regulated or down-regulated genes and the first proximal aERBS; (**b**) Workflow of the analysis performed to identify SE-associated lncRNAs regulated by apoERα; (**c**) Sub-network representing ERα ChIA-PET interactions involving *TFF1* (**top**) and *DSCAM-AS1* locus (**bottom**). The gene nodes are color-coded based on the log2FC of expression measured in the siERα RNA-Seq experiment. The nodes representing apoERα binding sites (aERBSs, diamonds) are color-coded according to their predicted chromatin states in HD MCF-7 from [27]. The links width is proportional to the number of ChIA-PET fragments confirming a long-range interaction; (**d**) Representative immunoblotting of p300, GATA3, FoxM1, CTCF, ERα and Hsp90 in MCF-7 cells grown in HD and transfected with siERα or siCTR; (**e**) Bar plot representing quantitation of p300, GATA3, FoxM1, CTCF and ERα protein level relative to Hsp90 protein level in MCF-7 cells grown in HD and transfected with siERα or siCTR; standard deviation of three biological replicates; p-value by unpaired t-test: *p<0.05; **p<0.01; (**f**) Bar plots reporting ERα fold enrichment over IgG signal by ChIP-qPCR at *AC099850.1*, *LINC00243*, *RP11-68L18.1* promoter and enhancer (SE-aERBSs) regions, in MCF-7 cells grown in HD and transfected with siERα or siCTR. SE-aERBSs showing an interaction with the associated lncRNA-promoter in the ERα-ChIA-pet experiment are indicated as SE-SE-aERBSs. Standard error of three biological replicates; p-value by unpaired t-test: *p<0.05. (**g**) Bar plots reporting CTCF fold enrichment over IgG signal by ChIP-qPCR at regions upstream to *DSCAM-AS1* E6-enhancer and downstream to *DSCAM-AS1* gene in MCF-7 cells grown in HD and transfected with siERα or siCTR (**left**) and treated for 45′ with 17β-estradiol (E2) or vehicle (veh) (**right**). Standard error of four biological replicates; p-value by unpaired t-test: *p<0.05.

Supplementary Figure 2. Analysis of *DSCAM-AS1* in breast cancer cell lines. (a) WashU genome browser view of *DSCAM-AS1* genomic locus reporting the SE coordinates predicted in different wild type or somatic fusions of BC cell lines from [30]. The coordinates of the MCF-7 aERBSs are also reported at top; (**b-c**) Bar plots reporting the relative expression of *DSCAM-AS1* and GREB1 (positive control) in a time-course experiment of 17β-estradiol (E2) or vehicle (veh) treatment in T-47D (**b**) and ZR-75.1 (**c**) cells; SD of three biological replicates; p-value by unpaired t-test: *p<0.05; **p<0.01; ***p<0.001; (**d**) WashU genome browser view of *DSCAM-AS1* genomic locus reporting the genomic signal profiles of ERα (red) and H3K4me3 (light blue) ChIP-Seq experiments performed on primary tumors from patients responding (Good Prognosis, GP) or not (Negative Prognosis, NP) to Aromatase Inhibitors (AIs) or Tamoxifen treatment. The coordinates of the predicted SE and aERBSs are reported at the top as black and red boxes, respectively. The E6 enhancer and the DSCAM-AS1 locus are highlighted. (**e**) *DSCAM-AS1* expression level in MCF-7 cells transfected with siCTCF or control siRNA before and after E2 treatment [43]. (**f**) Box plots reporting the normalized number of ERα ChIP-Seq reads counted at DSCAM-AS1 E6 enhancer (left) and promoter (right) considering data from patient responsive (Good prognosis, Good) or not (Negative prognosis, Negative) to Tamoxifen treatment (AI).
